# Calprotectin in Chronic Inflammatory Demyelinating Polyneuropathy and Variants—A Potential Novel Biomarker of Disease Activity

**DOI:** 10.3389/fneur.2021.723009

**Published:** 2021-09-13

**Authors:** Frauke Stascheit, Benjamin Hotter, Sarah Klose, Christian Meisel, Andreas Meisel, Juliane Klehmet

**Affiliations:** ^1^Department of Neurology, Charité — Universitätsmedizin Berlin, Corporate Member of Freie Universität Berlin, Humboldt-Universität zu Berlin, Berlin, Germany; ^2^NeuroCure Clinical Research Center, Charité — Universitätsmedizin Berlin, Corporate Member of Freie Universität Berlin, Humboldt-Universität zu Berlin, and Berlin Institute of Health, Berlin, Germany; ^3^Center for Stroke Research Berlin, Charité — Universitätsmedizin Berlin, Corporate Member of Freie Universität Berlin, Humboldt-Universität zu Berlin, and Berlin Institute of Health, Berlin, Germany; ^4^Department of Immunology, Charité — Universitätsmedizin Berlin, Berlin, Germany; ^5^Department of Immunology, Labor Berlin, Charité Vivantes GmbH, Berlin, Germany; ^6^German Myasthenia Gravis Society (Deutsche Myasthenie Gesellschaft, DMG), Bremen, Germany; ^7^Department of Neurology, Jüdisches Krankenhaus Berlin, Berlin, Germany

**Keywords:** calprotectin (S100A8/S100A9), serum neurofilament light chain, chronic inflammatory demyelinating polyneuropathy, CIDP variants, biomarker

## Abstract

**Background:** In chronic inflammatory demyelinating polyneuropathy (CIDP), there is an urgent need for biomarkers to monitor ongoing disease activity. Serum calprotectin (CLP) induces signaling pathways involved in inflammatory processes and has been shown to correlate with markers of disease activity in other autoimmune disorders. Thus, we wanted to study the potential value of CLP in comparison to serum neurofilament light chain (sNfl) to monitor disease activity.

**Materials and Methods:** Sera from 63 typical and atypical CIDP and 6 MMN patients with varying degrees of disease activity were analyzed in comparison with 40 healthy controls (HC) in a cross-sectional design. Association of CLP and sNfl levels with socio-demographics, disease duration, CIDP disease activity scale (CDAS), and impairment status [medical research council-sum score (MRC-SS), the inflammatory neuropathy cause and treatment disability score (INCAT-DS), grip strength, and maximum walking distance], patient-reported outcome (PRO) parameters [SF-36 questionnaire, Beck's depression index (BDI), and fatigue severity scale (FSS)], as well as treatment regime were investigated using uni- and multivariate analysis.

**Results:** CLP and sNfl levels were significantly higher in all CIDP patients compared to HC (*p* = 0.0009). Multivariate analysis adjusted for age and gender revealed that CLP acts as an independent predictor for CIDP and MMN. CLP was significantly associated with active disease course according to CDAS and correlated with MRC-SS, whereas sNfl correlated with parameters of disease impairment. There was no correlation with PRO, except for sNfl and the mental health composite score. Subgroup analysis revealed no differences between typical CIDP and atypical variants.

**Conclusions:** CLP was elevated in CIDP and variants and was associated with active disease course, whereas sNfl shows further potential as biomarker of axonal degeneration. Thus, CLP might be a suitable additive biomarker for measurement of ongoing inflammation, which is greatly needed to guide better patient care in CIDP.

## Introduction

Chronic inflammatory demyelinating polyneuropathy (CIDP) is a rare, acquired immune-mediated demyelinating neuropathy, characterized by strong heterogeneity in terms of clinical presentation, prognosis, and treatment response (1). Diagnosis is based on progressive or relapsing course over 2 months, electrophysiological evidence of peripheral demyelination, and response to immune-modulating therapies (2–4). Different autoimmune targets are likely to be relevant, as both autoreactive T-cell responses against myelin antigens (5, 6) and auto antibodies (Abs) against paranodal (7) and ganglioside proteins (8) have been shown to be involved in the immunopathogenesis of CIDP. Thus, not only misdiagnosis is a common problem, as only a subgroup of patients presents with detectable auto-Abs, but also monitoring disease activity in order to guide treatment management, as nerve conduction studies and electromyography do not adequately reflect functional disability, especially in severe disease courses (9, 10).

Calprotectin (CLP), a calcium-binding protein of the S100 family, performs various biological functions *via* interaction with Toll-like receptor 4 (11) and is mainly released by activated leucocytes triggering signaling pathways involved in inflammatory processes (12). Thus, CLP has been investigated in other autoimmune diseases, mainly inflammatory bowel disease (IBD) (13, 14) and rheumatoid arthritis (RA) (15), where CLP strongly predicted disease activity and treatment response. CLP leads to the release of pro-inflammatory cytokines (16, 17) and induction of auto-reactive CD8+ T cells (17), which play a key role in the pathogenesis of CIDP (18, 19).

There is increasing evidence that serum neurofilament light chain (sNfl), which is released upon neuronal injury (20), is increased in patients with CIDP and correlates with markers of disease severity (21–24). Nevertheless, the major limitation of sNfl is the lack of age-specific cutoff values (25, 26). In addition, there is an unmet need for serum biomarkers to differentiate disease activity from disease progression.

Hence, we wanted to investigate the potential additive value of CLP as a disease activity biomarker using a cohort of CIDP patients with different variants compared to healthy controls (HC).

## Materials and Methods

### Standard Protocol Approvals, Registrations, and Patient Consent

The study was approved by the ethics committee of the Charité-Universitätsmedizin Berlin (EA1/025/11). All patients gave written informed consent in accordance with the Declaration of Helsinki in its currently applicable form.

### Study Design

This is an exploratory, cross-sectional case–control study comparing CLP levels of CIDP and MMN patients as well as HC patients to assess the potential of CLP in comparison to sNfl for monitoring disease activity and impairment as measured by CIDP disease activity scale (CDAS) (27), clinical impairment status [medical research council-sum score (MRC-SS) (28), the inflammatory neuropathy cause and treatment disability score (INCAT-DS) (29), grip strength, and walking distance], PRO parameters [short form health survey (SF-36 questionnaire) (30), fatigue severity scale (FSS) (31), and Beck's depression inventory (BDI) (32)], disease duration, and treatment regime.

### Patients and Controls

This study was performed at the outpatient center for inflammatory polyneuropathies of Charité-Universitätsmedizin Berlin, Germany. Patients over the age of 18 years who fulfilled the definite, probable, or possible EFNS/PNS criteria (3) were included independent of disease duration and severity. Moreover, all patients were tested for onconeural as well as paranodal and ganglioside antibodies (Abs) and were screened for the presence of other autoimmune disorders. Overall, 63 CIDP patients were consecutively screened at the outpatient clinic between April 2015 and February 2021 and were further categorized in subgroups according to clinical phenotype [typical, pure sensory, multifocal acquired demyelinating sensory, and motor polyneuropathy (MADSAM), and distal acquired demyelinating symmetric polyneuropathy (DADS)] (3, 33), aGAAbs-status, and treatment regime (therapy naïve; maintenance treatment with corticosteroids, intravenous immunoglobulins, immunosuppressive therapies; remission). Socio-demographics (age, sex), disease duration, patient-reported outcome (PRO) measures [SF-36 (30), FSS (31), BDI (32)], and comorbidities were collected in a database. Exclusion criteria were age <18, the presence of other autoimmune diseases due to potential influence in CLP levels, diagnosis of neurodegenerative diseases [atypical and typical parkinsonian disorders, frontotemporal dementia, Alzheimer's disease, and amyotrophic lateral sclerosis (34)], central nervous inflammatory diseases (multiple sclerosis and autoimmune encephalitis), and traumatic brain injury due to potential influence on sNFL levels. Healthy voluntary controls were enrolled as a HC group (*n* = 40).

### Clinical Assessment

Patients were examined by the treating neurologist (JK, BH, and FS). Socio-demographics and current medication were documented. To measure the muscle strength on the level of impairment, the medical research council sum scale (MRC-SS) (28) was assessed. For the MRC-SS the following muscles had been tested on both sides: shoulder abduction, elbow flexion, wrist extension, index finger extension, hip flexion, knee extension, ankle dorsiflexion, and extension of the big toe. A sum score of 80 indicates a normal muscle strength. The adjusted inflammatory neuropathy cause and treatment disability score (INCAT-DS) (29) was used to assess the clinical disability in daily arm and leg mobility and has evolved as the most established primary outcome in clinical trials (35). Grip strength was measured by using the Martin Vigorimeter (36), and we assessed maximum walking distance. PRO was measured using the German version of the SF-36 questionnaire consisting of eight subscales and two summary scores—physical health composite score (PCS) and mental health composite score (MCS). Scores are ranging from 0 to 100, where higher scoring indicates a better quality of life. FSS was assessed to evaluate severity of fatigue (31) as well as BDI (32) to screen for depression.

In addition, we assessed treatment regime (corticosteroids, intravenous immunoglobulins, plasmapheresis, and immunosuppressive therapies) and treatment duration and further categorized according to CDAS (27) into active disease status and remission.

### CLP and sNfl Measurements

Blood samples were collected from patients with CIDP and HC, immediately centrifuged, and stored at −80°C until being analyzed in May and February 2021. Serum levels of CLP were measured using the fCAL turbo method^?^ on a COBAS 8000 semi-automated analyses (Bühlmann Laboratories AG, Schönenbuch, Switzerland) according to the manufacturer's protocol (37, 38). The fCAL turbo method? has been validated for accurate measurement of CLP levels in serum (37). sNfl concentrations were measured using SIMOA Nf-light kit^®^ in SR-S immunoassay analyzer, SIMOA^™^ (Quanterix Corp., Boston, MA, USA) according to the manufacturer's protocol (39). SIMOA Nf-light kit^®^ is an ultrasensitive paramagnetic bead-based enzyme-linked immunosorbent assay, is at least 125 times more sensitive than conventional immunoassays, and maintains a high analytical performance (40).

### Statistical Analysis

All statistical analyses were performed using Prism (version 9.0.1, GraphPad Software, San Diego, CA, USA) and SPSS (version 25; IBM, Armonk, NY, USA). Continuous data are presented as means and standard deviation (SD) or median and quartiles as appropriate, and categorical variables are presented as absolute frequencies and percentages. CLP and sNfL levels between typical CIDP, atypical variants, and HC were compared using non-parametric tests given that CLP and sNfl levels are not normally distributed. Correlations between CLP, sNfL levels, and clinical assessments were analyzed by Spearman correlation. Mann–Whitney *U* and Kruskal–Wallis tests were used to compare medians of non-parametric variables between two or more groups, and Fisher's *t*-test was used to compare parametric variables. A two-tailed *p* < 0.05 was considered statistically significant. Multivariate logistic regression analysis was performed to assess dependency of CLP and sNfl levels on age and gender.

### Primary Research Questions

Are CLP levels elevated in typical CIDP patients and atypical variants compared to HC and can elevated levels be used as a biomarker to monitor disease activity and PRO?

## Results

### Demographics and characteristics of CIDP and MMN patients

Altogether, we included 63 patients with CIDP and 40 HC (Table 1). Thirty-one patients suffered from typical CIDP (45%), and 32 (46%) suffered from an atypical variant. Of those with atypical variants, 9 patients suffered from pure sensory CIDP (14%), 14 (22%) from DADS, and 9 patients (14%) from MADSAM. Mean age was 63.7 years (SD 11.9 [IQR 55–73]); 60% (*n* = 38) were male. Median disease duration was 8.0 years (IQR 4.0–12.0). CIDP patients and HC did not differ in sex; however, atypical and MMN patients were more frequently male (75%, *n* = 24; 80%, *n* = 4). The majority of patients had an active disease activity status (81%, *n* = 51); 19% (*n* = 12) were in remission based on CDAS (27).

**Table 1:** Patient characteristics.

	**Total**	**Typical CIDP**	**Atypical CIDP**	**MMN**	**HC**	**Baseline statistic**
Demographics	63	31 (45%)	32 (46%)	6 (9%)	40	
**Sex**						
Male	38 (60%)	14 (45%)	24 (75%)	4 (80%)	17 (43%)	17 (43%) *p* = 0.0256^*^
Age at time point of sampling (years)	63.7 ± 11.9	59.6 ± 17.3	64.1 ± 10.9	62.2 ± 11.1	48.1 ± 8.5	48.1 ± 8.5 *p* <0.001^*^
Disease duration (years)	8.0 (4.0–12.0)	9.5 (3.3–16.8)	7.0 (4.0–10.0)	5.0 (3.8–14.8)		–
**EFNS/PNS criteria**						
Definite	33 (52%)	18 (58%)	15 (48%)	6 (100%)		–
Probable	30 (48%)	13 (42%)	17 (52%)	0 (0%)		
**Disease activity status**						
Active stabile	51 (81%)	26 (84%)	25 (78%)	6 (100%)		–
Remission	12 (19%)	5 (16%)	7 (22%)	0 (0%)		
**INCAT-score (SD)**						
Arms	1.4 (0.9)	1.6 (1.1)	1.5 (1.0)	2.0 (0.6)		–
LEGS	1.3 (0.7)	1.6 (0.9)	1.4 (0.8)	1.2 (0.8)		
**Grip strength KPA (SD)**						
Right arm	68.3 (23.3)	66.7 (27.6)	74.3 (19.2)	53.2 (19.5)		
Left arm	63.1 (26.9)	61.5 (28.4)	70.5 (21.5)	65.6 (25.7)		–
Medical research council (SD)	74.3 (4.6)	73.1 (9.1)	72.3 (7.6)	73.0 (6.1)		–
Max. walking distance in meters	1,000 (200–3,250)	1,000 (200–2,000)	1,000 (500–4,750)	5,500 (400–32,500)		–
**Median (IQR)**						
SF-36 TOTAL (SD)	78.4 (16.5)	79.2 (16.2)	77.7 (17.4)	68.5 (14.9)		–
SF-36 PSC (SD)	32.2 (10.2)	30.9 (6.9)	34.1 (11.8)	37.9 (14.6)		–
SF-36 MSC (SD)	44.4 (13.5)	47.1 (12.0)	42.3 (14.5)	30.5 (0.6)		–
FSS (SD)	4.6 (1.7)	4.8 (1.8)	4.5 (1.8)	5.3 (1.4)		–
BDI (SD)	12.3 (8.3)	8.4 (2.0)	12.3 (8.5)	19.3 (5.5)		–
**Treatment**						
Corticosteroids	8 (12%)	6 (19%)	2 (6%)	0 (0%)		–
IVIG	35 (56%)	17 (55%)	18 (56%)	5 (84%)		–
Plasmapheresis	4 (10%)	2 (7%)	2 (6%)	0 (0%)		–
AZT/MMF	4 (10%)	3 (10%)	1 (3%)	0 (0%)		–
Rituximab	3 (4%)	2 (7%)	1 (3%)	0 (0%)		–

*Data are mean (SD) and n (%) for the baseline variables and median (IQR) for disease duration. Disease duration is the time from diagnosis until baseline. AZT/MMF, azathioprine/mycophenolate mofetil; CIDP, chronic inflammatory demyelinating polyneuropathy; EFNS/PNS, European Federation of Neurological Societies/Peripheral Nerve Society; HC, healthy controls; INCAT, inflammatory neuropathy cause and treatment sensory sum score; IVIG, intravenous immunoglobulins; IQR, interquartile range; MMN, multifocal motoric neuropathy; MRC, medical research council sum score; SD, standard deviation; –, not applicable;*

* *statistical analysis was performed using Mann–Whitney U-test*.

### CLP and sNfl Is Independently Associated With CIDP and Variants

**Figure 1:**
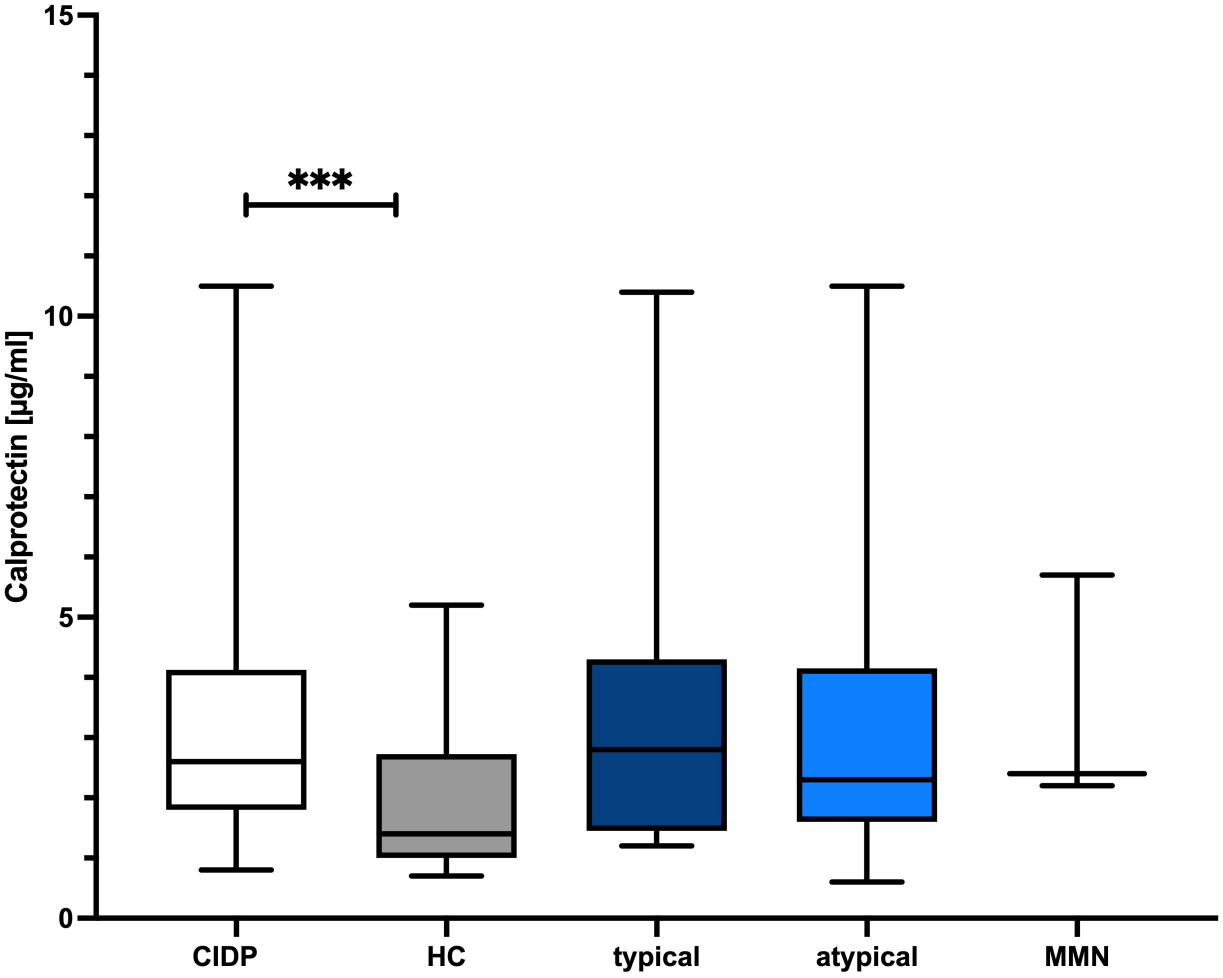
Comparison of CLP level in CIDP and HC. The boxplot bar represents median (min.–max.) of serum calprotectin (CLP) level in all CIDP patients (*n* = 63) and HC (*n* = 40). Median CLP levels were significantly increased in all CIDP patients regardless of subtype (*p* = 0.009) (typical: *n* = 31, atypical: *n* = 32, MMN *n* = 6). CIDP, chronic inflammatory demyelinating polyneuropathy; HC, healthy controls; min., minimum; max., maximum; MMN, multifocal motoric neuropathy; *n*, number of included study participants. ***p < 0.001, ****p < 0.0001.

**Figure 2:**
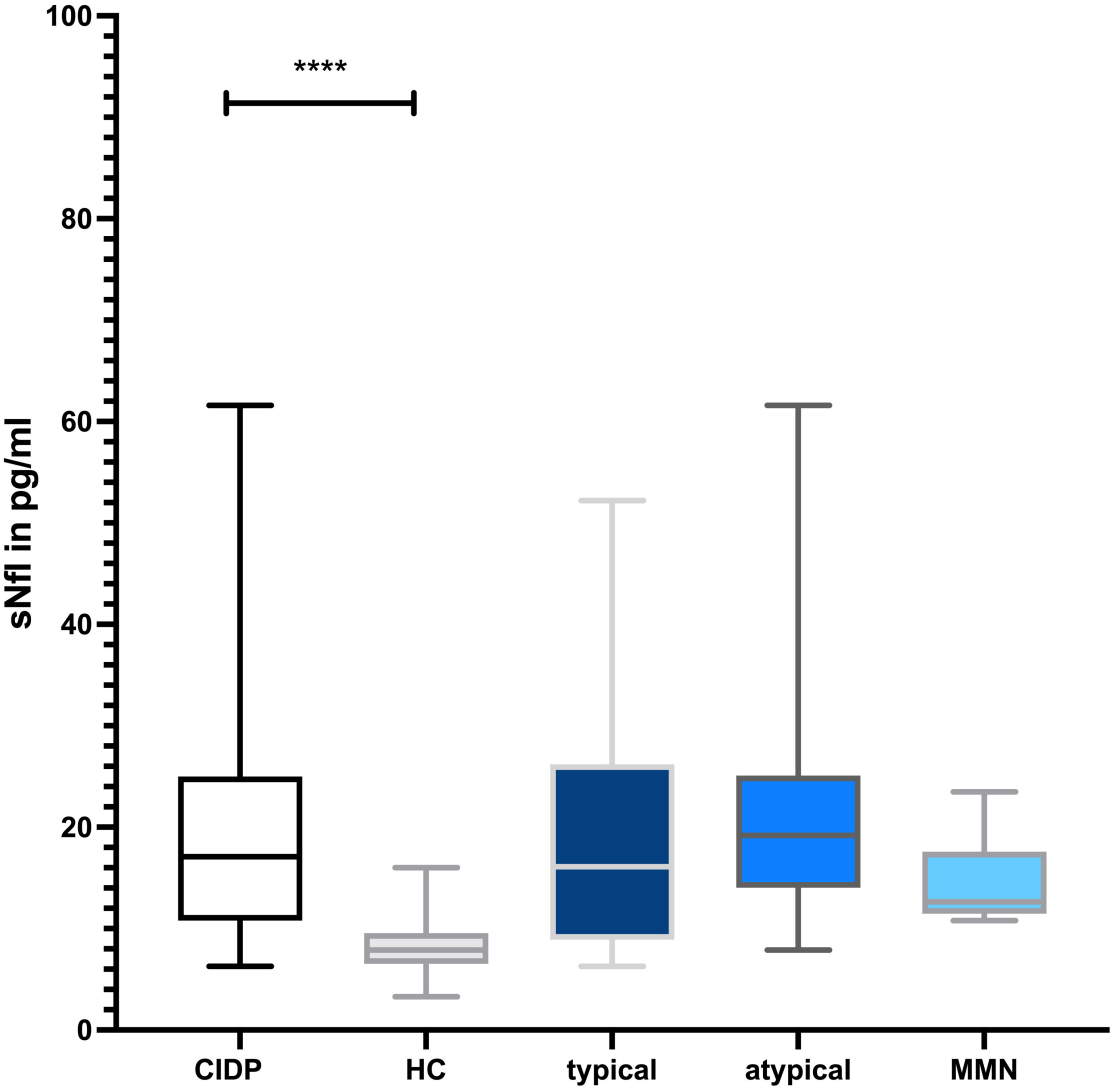
Comparison of sNfl level in CIDP and HC. The boxplot bar represents median (min.–max.) of serum neurofilament (sNfl) level in all CIDP patients (*n* = 63) and HC (*n* = 30). Median sNfl levels were significantly increased in all CIDP patients regardless of subtype (*p* < 0.001) (typical: *n* = 31, atypical: *n* = 32, MMN *n* = 6).

CLP levels were significantly higher in CIDP patients with a median of 2.6 μg/ml (IQR 1.8–4.1) compared to 1.4 μg/ml in HC (IQR 1.0–2.7*; p* = 0.0009, Mann–Whitney *U*-test; Figure 1). sNfl levels were significantly higher in CIDP patients with a median of 17.6 pg/ml (IQR 11.0–25.1) compared to 7.9 pg/ml in HC (IQR 6.5–9.5; *p* < 0.0001; Mann–Whitney *U*-test; Figure 2).

There was no significant difference in median CLP and sNfl levels between typical and atypical CIDP variants as well as MMN patients (*p* = 0.79 for CLP; *p* = 0.199 for sNfl, Kruskal–Wallis test). There was no correlation of CLP with age (*r* = 0.156, *p* = 0.136, Spearman correlation coefficient) and no association with gender (*p* = 0.299; Mann–Whitney *U*-test), whereas sNfl was highly associated with both age (*r* = 0.685, *p* < 0.001), and gender (*p* = 0.01; Mann–Whitney *U*-test).

Multivariate binary logistic regression models with backward stepwise inclusion to establish independence of age and gender were performed. In the model using sNFL to predict inflammatory neuropathies (*p* < 0.001), sNFL was the only remaining independent predictor, whereas the CLP model retained age and CLP (both *p* < 0.001) as independent predictor. Age was not distributed equally among the inflammatory neuropathies group and HC (Mann–Whitney *U*-test, *p* < 0.001).

### CLP Levels Associated With CDAS and MRC-sum Score

**Figure 3:**
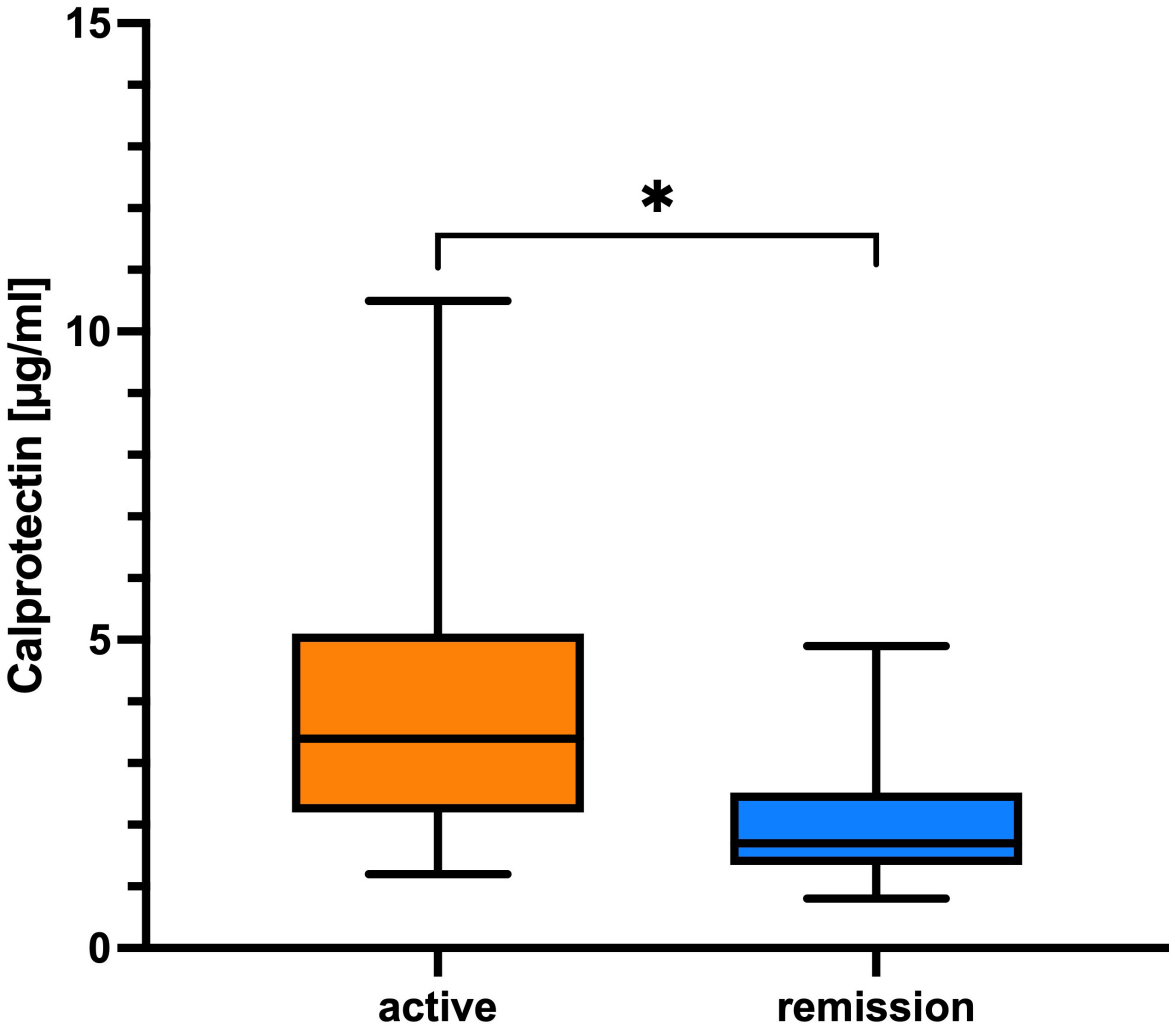
Comparison of CLP in regard to CDAS. The boxplot bar represents median (min.–max.) serum calprotectin level in regard to CIDP disease activity scale (CDAS) showing higher levels in patients with active disease (*n* = 51) in comparison to patients in remission (*n* = 12; *p = 0.025, Fisher's exact *t*-test).

CLP was significantly associated with active disease course (unstable active disease and active disease) based on CDAS definition (27) (*p* = 0.025, Fisher's exact *t*-test) (Figure 3) and correlated moderately with MRC-sum score (*r* = 0.35, *p* = 0.015; Spearman correlation coefficient), whereas sNfl correlated with all parameters of disease impairment as well as with disease duration (*r* = 0.32, *p* = 0.006; Spearman correlation coefficient) (Table 2).

**Table 2:** Correlation matrix for disease activity and impairment.

	**Serum CLP**	**Serum NFL**
Disease duration	*r* = 0.09, *p* = 0.47	*r* = 0.32, *p* = 0.006^**^
Disease activity status	*p* = 0.025^*^	*p* = 0.74
MRC sum score	*r* = 0.35, *p* = 0.013^*^	*r* = −0.42, *p* = 0.0008^***^
INCAT	*r* = 0.007, *p* = 0.95	*r* = 0.32, *p* = 0.009^**^
Grip strength	RIGHT ARM: *r* = 0.06, *p* = 0.673 LEFT ARM: *r* = 0.02, *p* = 0.88	RIGHT ARM: *r* = −0.34, *p* = 0.025*LEFT ARM: *r* = −0.46, *p* = 0.002^**^
Walking distance	*r* = 0.19, *p* = 0.167	*r* = −0.35, *p* = 0.004^**^

*Correlation matrices of parameters of clinical disease activity and impairment of serum calprotectin (CLP) and serum neurofilament light chain (sNfl) in CIDP patients and variants. Values are Spearman r;*

* *p < 0.05;*

** *p < 0.01;*

*** *p < 0.001 or tested using Kruskal–Wallis test (disease activity status scale). MRC, medical research council sum score; INCAT, inflammatory neuropathy cause and treatment sensory sum score*.

### CLP and sNfL Levels Are Not Associated With Patient-Reported Outcomes

Except for SF-MSC in correlation with sNfl (*r* = 0.32, *p* = 0.044; Spearman correlation coefficient), we found no association of CLP or sNfl in regard to PROs (FSS, BDI, and SF-PSC).

### sNfL Levels and Treatment Regime

Neither CLP nor sNfl did significantly differ in regard to treatment regime (*p* = 0.643 and *p* = 0.5, respectively; Kruskal–Wallis test).

## Discussion

This cross-sectional case–control study demonstrates for the first time that CLP is significantly increased in CIDP patients with different variants in comparison to HC. To emphasize, CLP was associated with active disease course as defined by CDAS and correlated with the MRC-SS. Thus, CLP might be of interest as an additional serum biomarker in the context of active CIDP. We also confirmed previous study results of sNfl being increased in CIDP patients correlating with parameters of clinical impairment. For emphasis and in order to reflect real-world-setting, we additionally examined the relationship of sNfl in regard to clinical characteristics and PRO parameters. The latter has not been investigated in other studies so far (21–24), although we found no significant relationship.

Despite recent advances in biomarker research for CIDP, nodal, paranodal, and anti-ganglioside antibodies (aGAAbs) can be detected only in a small proportion of patients (7, 8). For the larger CIDP population, diagnostic and prognostic blood biomarkers are still an unmet need. Even more important, there is a lack of objective, disease activity parameters to guide treatment response, especially in patients under maintenance therapy.

A recent report showed increased pathologic spontaneous activity to predict a more severe course of disease (9). Nevertheless, whether nerve conduction studies correlate with functional disability is still under discussion and requires external validation (10). Furthermore, electromyography is less readily available than a laboratory analysis, enabling repeated sampling and possibly allowing conclusions on the current disease activity.

CLP leads to induction of auto-reactive CD8+ T cells, IL-17 (41), and other pro-inflammatory cytokines like IL-1β. The imbalance of inflammatory cytokines and auto-reactive CD8+ T cells are crucially involved in the pathogenesis of CIDP and plays a central role. IL-17 was reported to be increased in peripheral blood mononuclear cell and cerebrospinal fluid of active CIDP in comparison with remitting CIDP or to other non-inflammatory neurological diseases (42). In addition, in a recent mouse model, auto-reactive CD8+ T cells were found in diseased nervous tissues, thus hinting further to a critical role in the pathogenesis and disease activity in CIDP (18). These results might explain the significant elevated CLP level in CIDP patients with disease activity according to CDAS, which we could not observe for sNfl. In addition, CLP correlated positively with MRC-SS, suggesting lower levels of axonal degeneration and persistent clinical impairment as in early phase of disease as well as in good treatment responders. This important finding relates to studies of CLP in IBD and RA, where CLP is routinely used as a disease activity marker (14, 43). CLP is a sensitive marker of inflammation mainly reflecting different degrees of disease activity and thus may guide treatment decisions, as proposed in other conditions like IBD (13) and RA (15, 44). In addition, CLP has the potential to serve as a possible diagnostic biomarker to differentiate inflammatory neuropathies from non-inflammatory neuropathies, as only a small percentage of CIDP patients present with nodal, paranodal, and aGAAbs. Future studies should therefore include non-inflammatory polyneuropathies as a diseased control group to further examine the potential diagnostic role of CLP in CIDP.

Our study confirms previous findings of elevated sNfl levels in patients with CIDP (21–24), although direct comparison of sNfl levels between studies should be done with caution, as methods of sNfl measurement vary. Hayashi et al. (23) found significantly elevated sNfl levels in 11 treatment naïve CIDP patients, which decreased after 1 month of IVIG therapy. Similar data were obtained by van Lieverloo et al., who found elevated sNlf levels in 29 patients starting induction therapy (21). However, in patients on maintenance therapy and in remission, increased sNfl levels were only infrequently found and did not differ from HC (21), which is in contrast to our results showing significant increased levels despite therapy. Mariotto et al. (22) and Fukami et al. (24) found a significant correlation of sNfl with disability using the overall neuropathy limitation in 12 CIDP patients and 58 CIDP patients, respectively. Latest study results are in line with our findings. Thus, the correlation of sNfl with disease impairment needs confirmation in future studies.

Neither CLP nor sNfl was significantly correlated with PRO, except for a weak correlation with the mental health composite score. Hence, CLP and sNfl objectively reflect ongoing clinical disease activity and impairment, although data on quality of life predictors in CIDP are still scarce. There is evidence that patients with more severe motor disability, short disease duration, and fatigue have a higher risk of worse quality of life (45). Thus, the relationship between PRO and blood biomarkers like CLP and sNfl should be investigated in larger, independent cohorts, since in other autoimmune diseases like IBD or multiple sclerosis, CLP and sNfl were shown to be a good predictor of health outcomes (46–48).

The major limitation of sNfl as a biomarker in neurological diseases is the lack of age-specific cutoff values, as sNfl levels in blood are age dependent increasing by an average of 2.2% per year in HC (25, 26). The reasons for this age-dependent increase might be mainly due to physiological age-dependent neuronal loss. In line with studies of sNfl in central neurodegenerative and neuroinflammatory diseases, establishing cutoff values over a wide range of ages for sNfl levels is critical to develop an individual, longitudinal biomarker, for assessment of disease severity in CIDP. In addition, sNfl correlated significantly with all parameters of disease impairment and disease duration hinting to its role to act as a biomarker of ongoing axonal degeneration.

In contrast, CLP is not an age-dependent biomarker (49), and we could not observe a correlation to age in our cohort. In addition, CLP is stable at room temperature and routinely measurable, which makes it an ideal candidate biomarker.

This study was conducted with a well-defined patient cohort with high diagnostic certainty. Nevertheless, there are several limitations to our pilot study. First, the cross-sectional cohort was rather small considering the subgroup analysis. In addition, the main proportion of included patients was not treatment naïve and heterogeneous in regard to treatment and disease duration. Nevertheless, this diversity reflects the typical makeup of a specialized clinic for inflammatory neuropathies. We have not included electrophysiological proxy parameters of axonal damage, mainly due to most included patients not having a nerve conduction study or myography within a short time of blood sample collection. We furthermore cannot rule out selection bias, as we recruited a convenience sample to perform this exploratory study. Our group of HC is significantly younger than the CIDP patients. We tried to match for age and gender as good as possible, which was often limited by confounding comorbidities increasingly prevalent with higher age. We accepted this convenience sample in light of evidence from many other studies and meta-analyses examining CLP in other autoimmune diseases like RA (15) or IBD (13, 14), that CLP is not influenced by age and tried to correct for its influence with multivariate analyses. Nevertheless, future confirmation studies will need to further control for this possible confounder with a much closer matched control group. In addition, in this pilot study, we have not included controls with non-inflammatory polyneuropathies as we first wanted to examine whether CLP is increased in CIDP patients and variants. Nevertheless, this study provides promise that CLP might also be of diagnostic value to discriminate inflammatory polyneuropathies from non-inflammatory polyneuropathies, which has to be examined in future studies.

In conclusion, this explorative cross-sectional study shows that CLP is significantly elevated in CIDP patients compared to HC and is associated with active disease course. In addition, this study confirms previous findings of elevated sNfl levels in CIDP, which correlate with disease impairment and disease duration, thus confirming sNFL as a potential marker of axonal degeneration. There is an unmet need of a validated, non-invasive biomarker in order to guide treatment decisions. CLP might potentially be a candidate additive biomarker for assessment of disease activity. Further multicentric, longitudinal investigations are strongly needed to determine the potential utility of CLP for a better patient care in CIDP.

## Data Availability Statement

The raw data supporting the conclusions of this article will be made available by the authors, without undue reservation.

## Ethics Statement

The studies involving human participants were reviewed and approved by Charité-Universitätsmedizin Berlin (EA1/025/11). The patients/participants provided their written informed consent to participate in this study.

## Author Contributions

FS: designed and conceptualized the study, had a major role in acquisition of data, analyzed and interpreted the data, drafted the manuscript for intellectual content, and takes full responsibility of the integrity of the data analyzed. BH: conceptualized the study, analyzed and interpreted the data, and revised the manuscript for intellectual content. SK: acquisition of data and revised the manuscript for intellectual content. CM: laboratory analysis and revised the manuscript for intellectual content. AM: interpreted the data and revised the manuscript for intellectual content. JK: designed and conceptualized the study, had a major role in acquisition of data, revised the manuscript for intellectual content, and revised the manuscript for intellectual content. All authors contributed to the article and approved the submitted version.

## Conflict of Interest

BH reports compensation for the conduct of clinical trials by argenx BVBA. CM is employed by Labor Berlin, Charité Vivantes GmbH. AM received speaker honoraria from Alexion, GRIFOLS, and Hormosan. He received honoraria from Alexion, MorphoSys, and Vitaccess for consulting services and financial research support from Octapharma and Alexion. AM is chairman of the medical advisory board of the German Myasthenia Gravis Society. JK received speaker honoraria from Merck Serono, Bayer, Biogen, Roche, Sanofi Aventis, Grifols, and Octapharma. She received honoraria for consulting services from Biogen, Merck Serono, Roche as well as financial research support from Octapharma and Grifols. The remaining authors declare that the research was conducted in the absence of any commercial or financial relationships that could be construed as a potential conflict of interest.

## Publisher's Note

All claims expressed in this article are solely those of the authors and do not necessarily represent those of their affiliated organizations, or those of the publisher, the editors and the reviewers. Any product that may be evaluated in this article, or claim that may be made by its manufacturer, is not guaranteed or endorsed by the publisher.
